# Supplementary figures and images for: Ubiquitin variants potently inhibit SARS-CoV-2 PLpro and viral replication via a novel site distal to the protease active site
Source: PLoS Pathog. 2022 Dec 22;18(12):e1011065. doi: 10.1371/journal.ppat.1011065 (PMC9822107; doi:10.1371/journal.ppat.1011065)

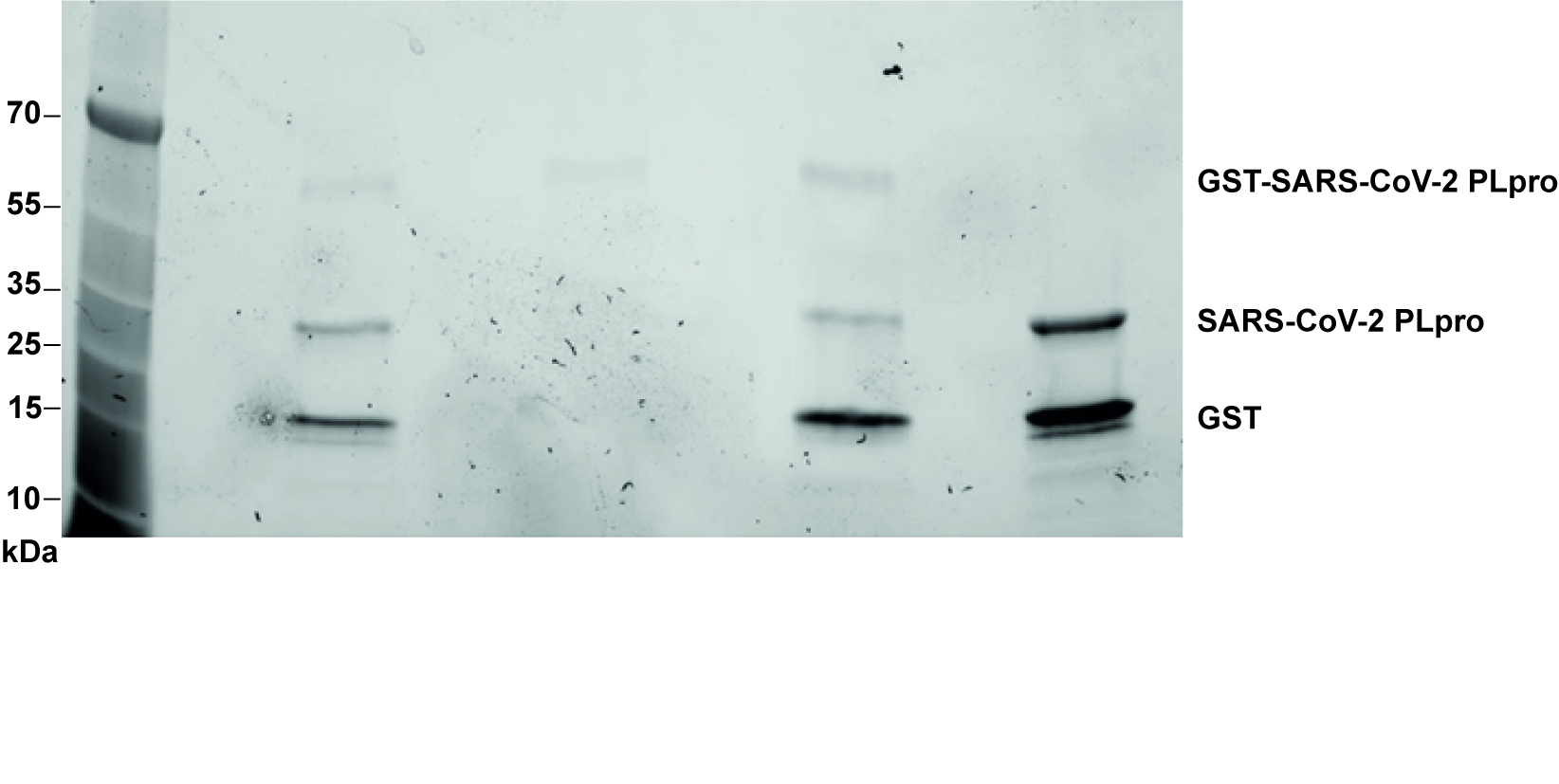

Supplement: S1 Fig — Purified GST-SARS-CoV-2 PLpro fusion protein has a mass of ~60 kDa according to SDS-PAGE and could be readily cleaved into free GST (~25 kDa) and SARS-CoV2 PLpro (~35 kDa) using HRV3c Precision Protease. The lane on the far left contains the molecular weight ladder and masses are shown on the left. Remaining lanes are various optimizations of the cleavage assay, with optimal cleave results shown in the far right lane. (TIF) [file ppat.1011065.s001.tif]

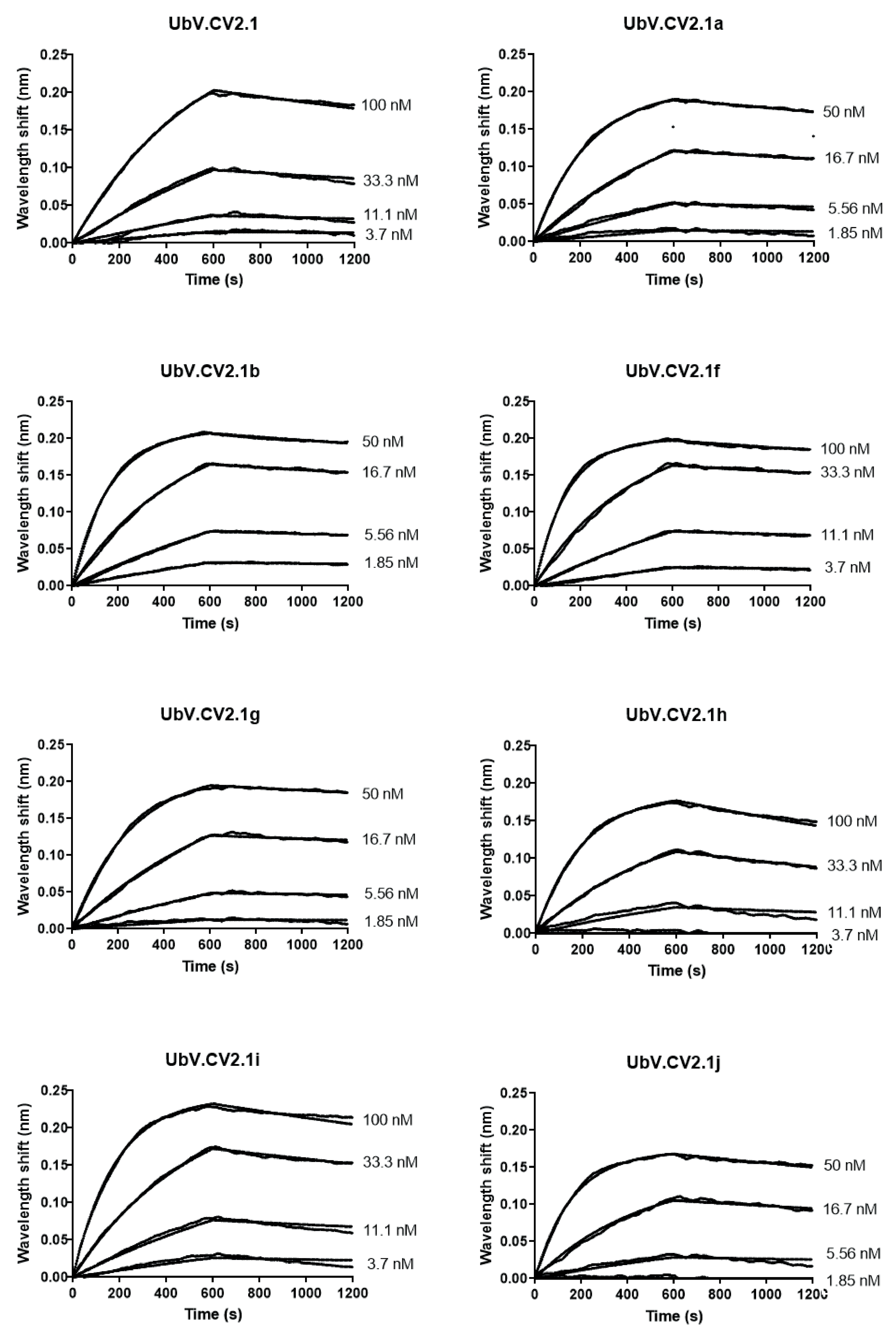

Supplement: S2 Fig — BLI curve fits of soluble UbVs with immobilized SARS-CoV-2 PLpro. Curves are shown for the parent UbV.CV2.1 and some of its optimized variants. (TIF) [file ppat.1011065.s002.tif]

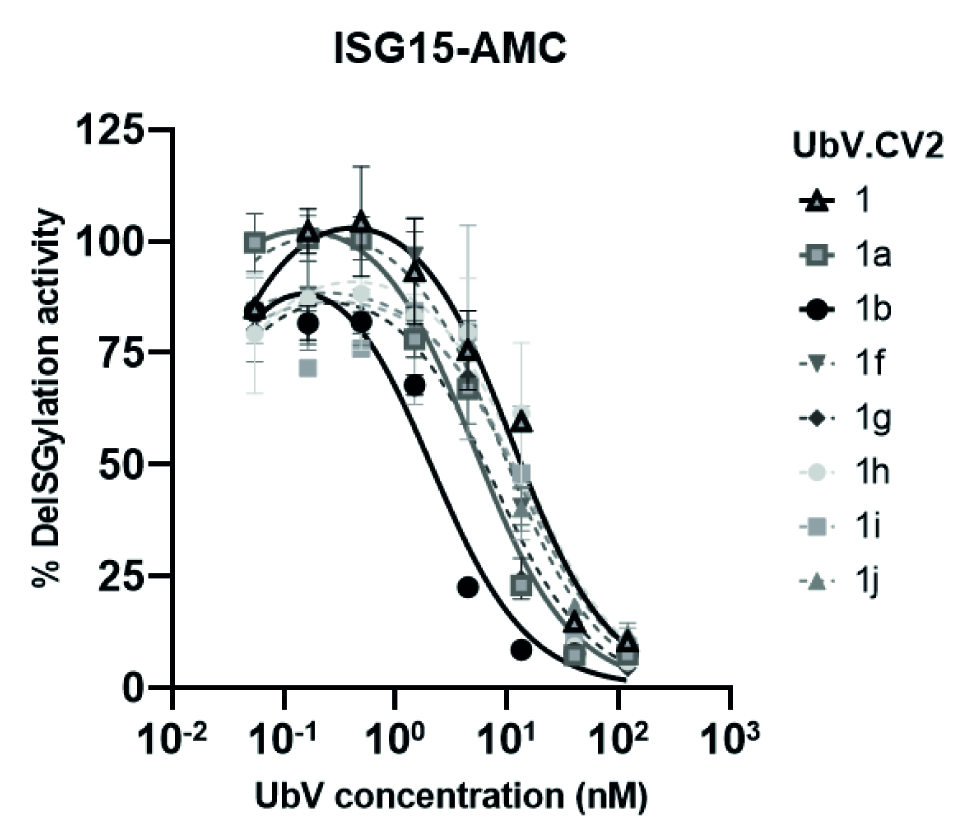

Supplement: S3 Fig — Inhibition of SARS-CoV-2 PLpro by the cognate UbVs shown as dose-response curves using ISG15-AMC as a substrate. The IC50 value was determined as the concentration of UbV that reduced enzyme’s activity by 50%. (TIF) [file ppat.1011065.s003.tif]

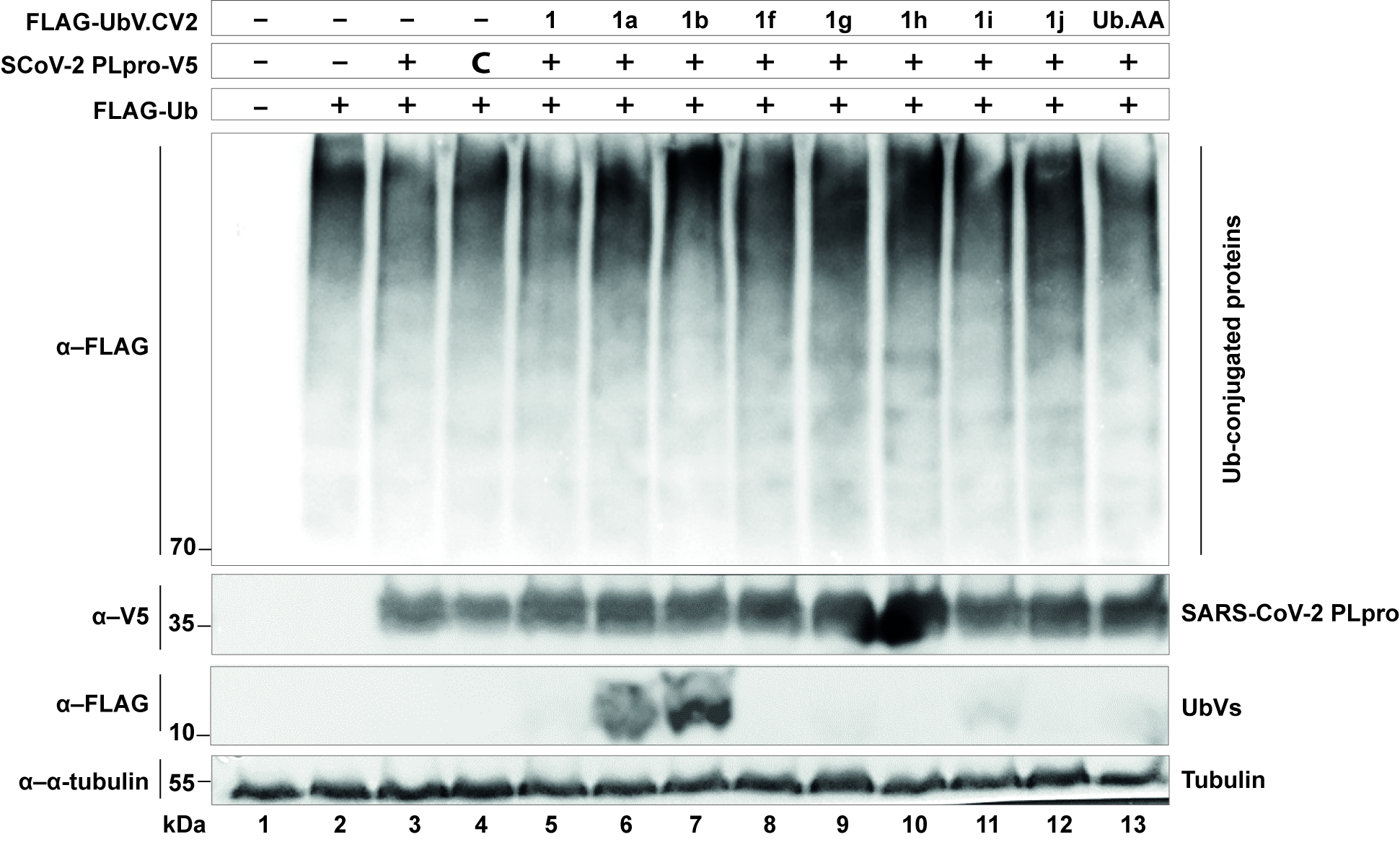

Supplement: S4 Fig — Inhibition of the deubiquitinating activity of SARS-CoV-2 PLpro by UbVs, visualized by co-transfection of plasmids encoding FLAG-Ub.WT, SARS-CoV-2 PLpro-V5 (wildtype or catalytic mutant ‘C’) and FLAG-UbVs into HEK293T cells. Lysates were collected 24 hours post transfection and were subjected to western blot analysis. DUB activity of PLpro is shown by the removal of FLAG-Ub from cellular substrates. Co-expression of some of the UbVs causes inhibition of PLpro DUB activity and ubiquitination of cellular proteins. (TIF) [file ppat.1011065.s004.tif]

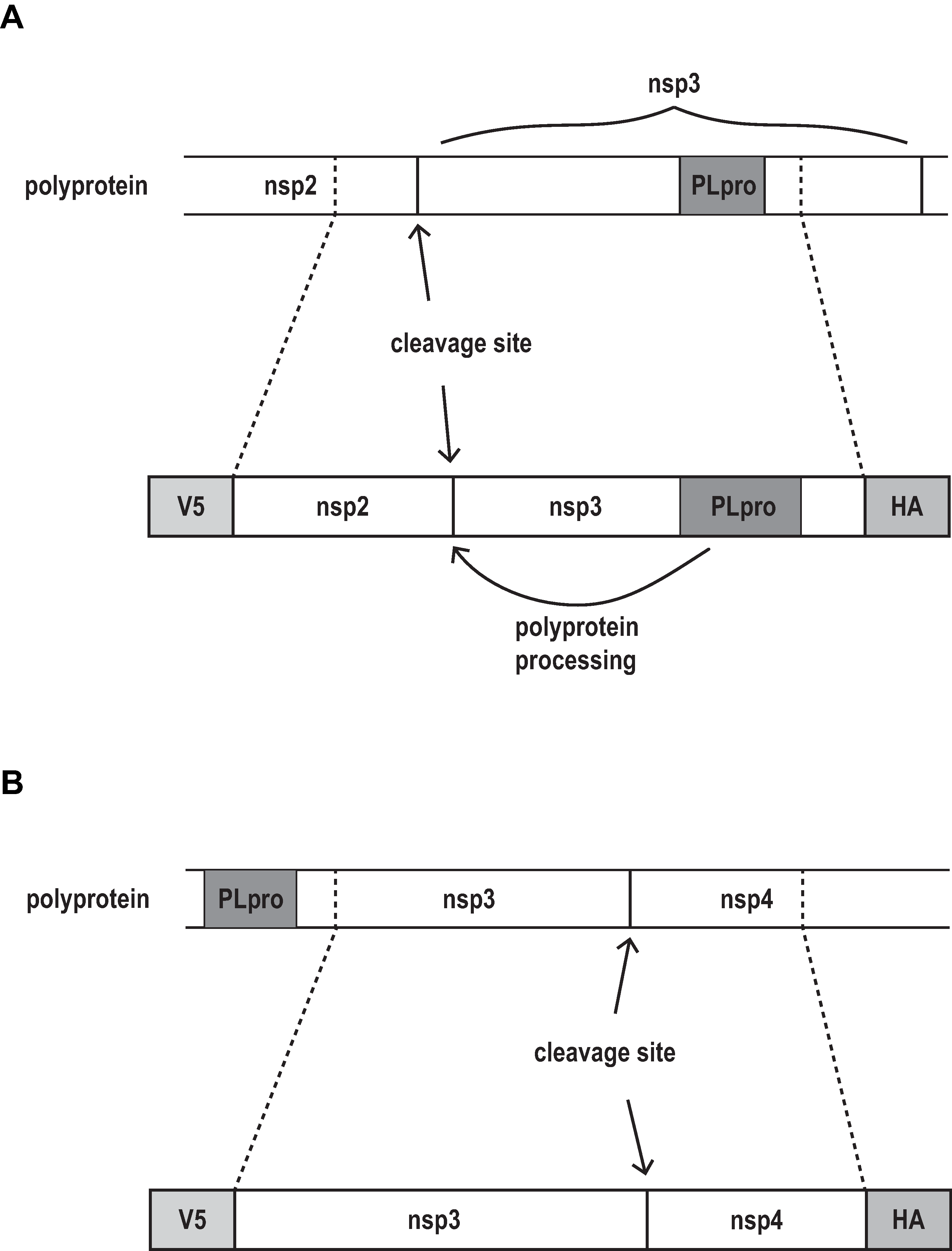

Supplement: S5 Fig — (A) Illustration of the construct used for the in vitro cleavage assay as shown in Fig 3A. The construct spans part of nsp2, part of nsp3 including the PLpro domain, as well as tags on either side for easy identification. (B) Representation of the constructs used for the polyprotein cleavage assay shown in Fig 3B. The construct spans part of nsp3 (excluding the PLpro domain), and part of nsp4, as well as tags on either side. (TIF) [file ppat.1011065.s005.tif]

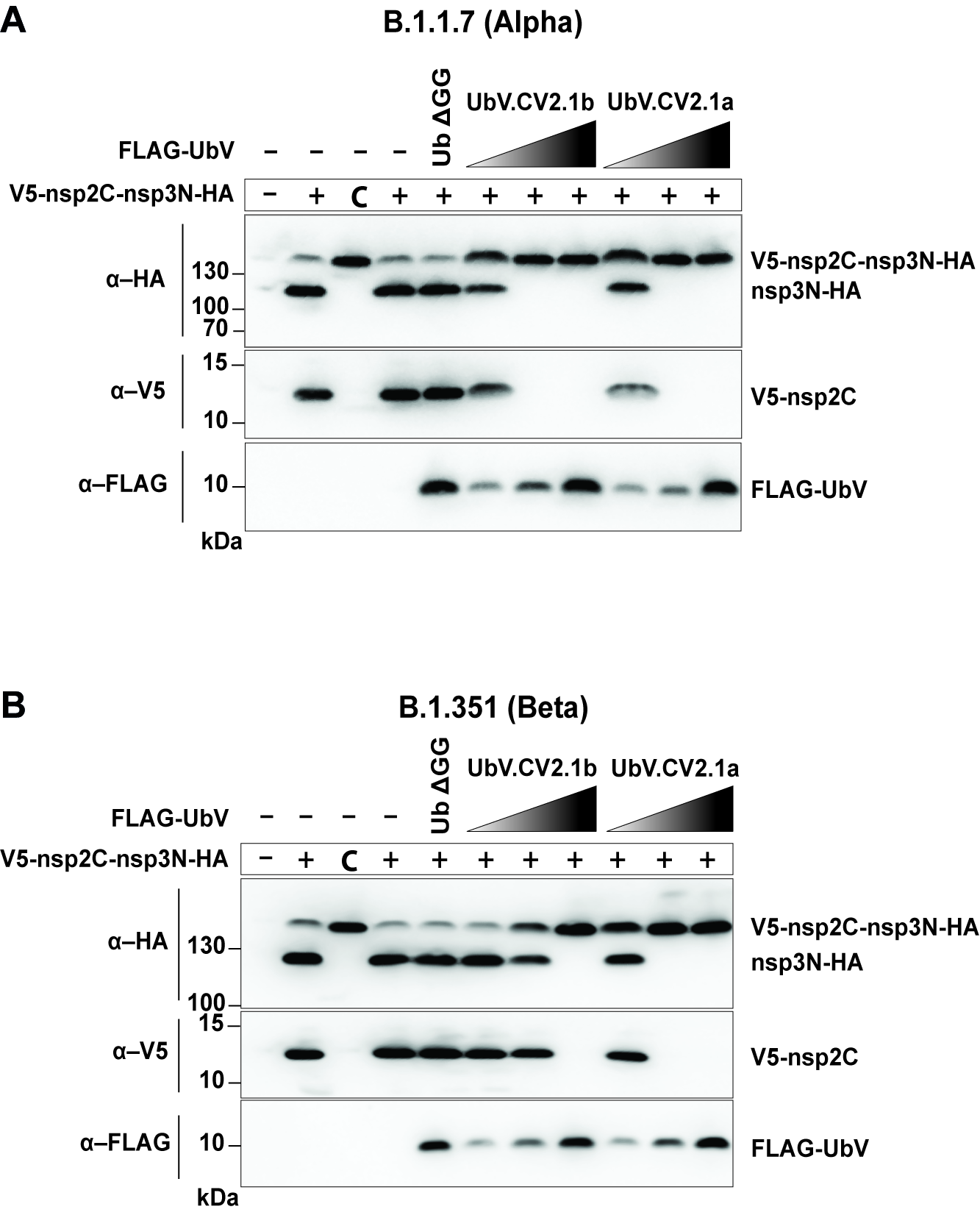

Supplement: S6 Fig — In vitro proteolytic cleavage capability of SARS-CoV-2 PLpro from the alpha (A) or beta (B) variants was assessed in the presence of the UbV.CV2.1a and 1b at different concentrations. Construct encoding N-terminal V5-tagged and C-terminal HA-tagged nsp2C-nsp3N (including the PLpro domain), V5-nsp2C-nsp3N-HA, was transcribed and translated in vitro in the presence of UbV.CV2.1a or 1b (with increasing doses) for 2 hours. Proteolytic cleavage activity was assessed by western blotting to detect the presence of N-terminal V5-tagged nsp2C and C-terminal HA-tagged nsp3N cleavage products. (TIF) [file ppat.1011065.s006.tif]

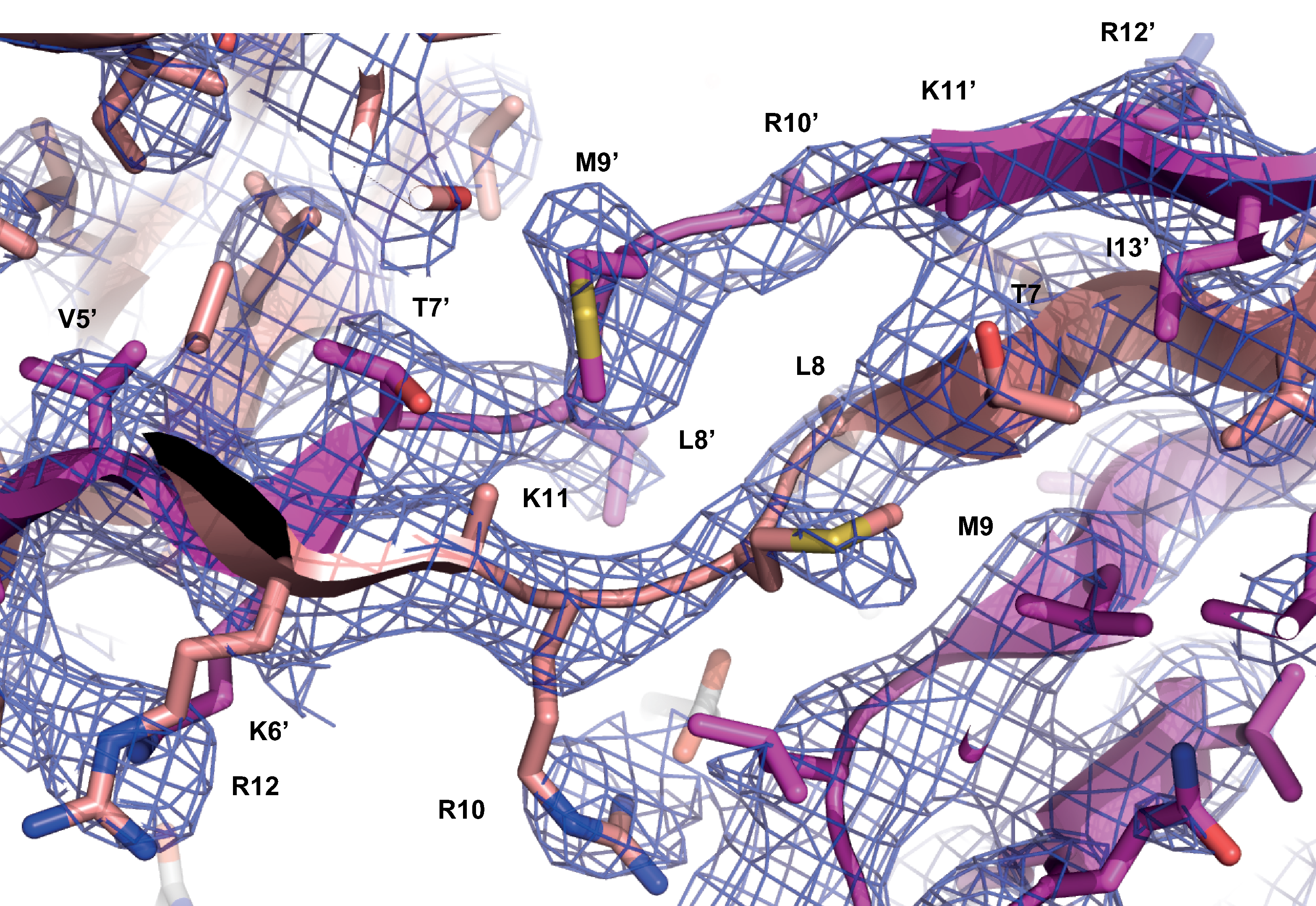

Supplement: S7 Fig — Electron density from a simulated annealing omit map generated by omitting residues 7–11 of all the UbV chains (chains E-L) from the model. The simulated annealing omit map was generated using Phenix (ref), electron density then was plotted up to 2.5 A around the UbVs (chain E and I of the model), and contoured at sigma = 1.2. The resulting electron density in chains E and I around this region shows 2 continuous beta strands through this region, indicative of a swapped dimer. (TIF) [file ppat.1011065.s007.tif]
