# Supplementary material for: Ubiquitin variants potently inhibit SARS-CoV-2 PLpro and viral replication via a novel site distal to the protease active site
Source: PLoS Pathog. 2022 Dec 22;18(12):e1011065. doi: 10.1371/journal.ppat.1011065 (PMC9822107; doi:10.1371/journal.ppat.1011065)
Supplement: Attachment — Submitted filename: 221207_Rebuttal PLoS Path dec 2022.docx [file ppat.1011065.s008.docx]

Dear Editor,

We would like to thank the reviewers and the editors for their time and their useful comments, as well as their general positive assessment of our work in their summaries. We have adapted the manuscript according to the concerns and suggestions of the reviewers. Please find below a point-by-point response, and explanation of the changes we have made. Referral to text and figures in the manuscript will be based on the revised version which contains tracked changes.

**(Part II – Major Issues)**

*Reviewer #1: 1. Figure 3 documents the reduction in viral RNA and pfu/ml at 24 and 48 hours post-infection and western blots showing the absence of M protein in UbV expressing cells. The results are consistent with UbV.CV2.1a impairing virus replication, but this does not show the target of this inhibition in the virus infected cells. One way to do this would be to infect cells at a very high MOI (perhaps using Vero-ACE2-TMPRSS2 expressing cells) and look at early times after infection (3-4 hrs) for the production of the ORF1a polyprotein products by western blot. The 3CLpro should be active and cleaving the downstream region of the polyprotein, but in the presence of the Ub variant, PLpro would be inhibited, generating a nsp1-2-3 polyprotein. This experiment would directly test if UbV.CV2.1a is directly blocking processing by PLpro . Alternatively, the Ub variant might be affecting virus entry or translation in some unexpected way, so it is important to determine if SARS-CoV-2 can enter and translate RNA into the ORF1a polyprotein in the presence of UbV.CV2.1a expression. Alternatively, this issue about what exactly UbV.CV2.1a is targeting in virus-infected cells could be part of the discussion.*

We agree with Reviewer 1 that it would be insightful to verify the exact mode-of-action of the UbV molecules during infection. The reviewer suggests to examine whether an uncleaved nsp1-2-3 polyprotein could be detected upon SARS-CoV-2 infection in cells expressing the Ub variant. We agree that it would fortify our story if we were able to visualize this product. However, as we showed in figure 3C and D, we have looked at the production of mature nsp1 and nsp3, which were not present in cells expressing the UbV. Whilst interpreting these blots, we paid special attention to the presence of higher molecular weight products, which would suggest a nsp1-3 polyprotein, but we were not able to detect these. We believe that because of the inhibitory activity of the UbV, not enough of the viral proteins is produced at all to be detected on western blot. This is because inhibition of polyprotein cleavage by PLpro in the beginning of the infection will very likely also hamper the functionality of the main protease (3C-like protease) and therefore the production of functional subunits downstream in the polyprotein, such as the polymerase. In turn, this would mean that not enough mRNA for the production of viral 1a/b polyprotein can be synthesized due to the lack of these functional downstream proteins, leading to the efficient inhibition of the infection as a whole, and illustrating the very high potency of PLpro as an antiviral target. The important regulatory function of the first protease in the nidovirus polyproteins (PLpro for the coronaviruses, PLP (in nsp2) for the arteriviruses, family members in the same order of nidoviruses) has been thoroughly investigated for equine arterivirus (EAV) in the past (for example Wassenaar AL, et al.. Alternative proteolytic processing of the arterivirus replicase ORF1a polyprotein: evidence that NSP2 acts as a cofactor for the NSP4 serine protease. J Virol. 1997 Dec;71(12):9313-22. doi: 10.1128/JVI.71.12.9313-9322.1997. PMID: 9371590; PMCID: PMC230234.). This data showed that if the first protease in the polyprotein is inhibited, the cleavage of the rest of the polyprotein by the second protease (Mpro/3C-like protease in coronaviruses) is also hampered. This particular effect has not been studied in detail for coronaviruses as far as we are aware, but the fact that we cannot detect functional polyprotein subunits nor polyprotein fragments upon the presence of Ub variants, fits with the idea that inhibition of PLpro’s function as a polyprotein cleaving protease (as can be shown in biochemical experiments presented in Fig. 3) and as a regulatory subunit strongly inhibits the correct processing of the rest of the replicase polyprotein, and thereby the infection as a whole. We cannot formally exclude that the Ub variant inhibits other processes during infection, but since our biochemical experiments clearly show the inhibition of the PLpro functions in the presence of Ub variant (Figs 2, Fig 3 and Fig S4) we believe the antiviral effect of the UbV is based on these strong effects on PLpro itself, and this to us strongly suggests that this explains the antiviral strength of UbV-mediated PLpro inhibition. We have added a short explanation of the expected effects of PLpro inhibition during infection to enrich the discussion starting at line 389 in the revised manuscript.

*Reviewer #2:*

*General comments:
The study is well-conducted with in vitro experiments combined with cell-based experiments and also in those infected with live virus. The reduction in viral titres in the presence of the UbV is significant. However, while the authors describe these Ub variants as potential antiviral therapeutic molecules, it is not clear how efficiently they can be delivered to cells. I have very few criticisms on this study; only a few minor comments.*

Reviewer 2 commented on the delivery of the UbVs into cells. We are aware of the difficulty of introducing protein products into cells and have commented on some possibilities to achieve this in the context of their potential use as antiviral agents in the discussion (starting at line 382). However, we consider any experiments pertaining to this to be outside the scope of this paper.

*Specific comments:
Given that the binding of the UbV appears to be equivalent for PLpro from CoV1 and CoV2, it might be useful to show whether CoV1 is inhibited as well.

Along the same lines, the activity of CoV2 and CoV1 PLpro have been shown to be different in their ability to hydrolyse ubiquitin modifications and ISG15 modifications (Munnur et al Nat Imm 2021, Shin et al Nature 2020). The loss of ISGylation (but much less so for ubiquitylation) is very easily visible in lysates from SARS-CoV2-infected or PLpro expressing cells, which has implications in immune subversion, even in the absence of a productive viral lifecycle (Munnur et al, Nat Imm 2021). It would therefore be important and easy to measure bulk ubiquitylation and ISGylation in these cells expressing the Ub variants and PLpro (and IFN-treated) or infected with virus, to test whether these profiles are affected.*

*Similarly, only inhibition of the deISGylase activity of PLpro has been shown with the UbVs; could the authors show whether the UbVs also affect DUB activity of the PLpro?*

Reviewer 2 suggests to look more closely at the deubiquitinating and deISGylation activities of PLpro and whether the UbV would be able to modulate these functions. We have, therefore, performed a DUB and a deISGylation assay using some of the UbV hits that resulted from the phage-library screen. We have added the resulting western blots from the deISGylation assay as a new figure (now figure 2) and have added the appropriate lines to the text describing the experiments and their outcomes (starting at line 164 in revised manuscript). As PLpro’s DUB activity is not as efficient as its deISGylation activity, the inhibitory effect of the UbVs is not as clear in this assay and therefore we have added the corresponding DUB assay blots as a supplemental figure (Fig S4). Furthermore, the reviewer requested data on the UbV activity against SARS-CoV (1) PLpro. We have therefore repeated similar DUB and deISGylation experiments, except with SARS-CoV PLpro instead of SARS-CoV-2 PLpro. These results showed that the UbVs do not have activity against SARS-CoV PLpro. We have therefore not included a figure of this in the manuscript, but have provided the corresponding blots below to answer the reviewers inquiry (A is a deISGylation assay, B a DUB assay). In addition, we have decided not to research this possible effect any further in the context of this paper, but it will be an interesting next step for further research.

*Proteolytic activity of PLpro is affected to same extent in PLpro mutant and UbV.CV2.1a/b (Fig 2A and 2B) - how does the distal binding of UbVs to PLpro impart the same level of inhibition as point mutation in active site for PLpro mutant – can authors speculate using structural information?*

The reviewer raises an interesting point about polyprotein (nsp2-3 or nsp3-4) cleavage being affected to the same extent by the UbVs (that bind distally) compared to the use of the catalytic mutant: we believe that this comes down to the steric hindrance performed by the UbV when it binds to PLpro in the distal site. Because of this binding, the pocket that harbours the catalytic site is no longer accessible for the substrate and while not directly binding the catalytic site, the UbV therefore does inhibit PLpro from binding and cleaving the polyprotein. To clarify this, we have adjusted the discussion in what is now line 376.

*It would be useful if the authors could comment on whether these variants would be able to equally inhibit PLpro from later viral variants of concern, based on the evolution of mutations in PLpro and their predicted structures.*

Reviewer 2 raises the question whether the UbVs would be able to inhibit PLpro of variants of concern. We have tested this using a polyprotein cleavage assay as shown in figure 3A to examine how UbVs affect PLpros encoded by the alpha and beta strains of SARS-CoV-2. The results from these experiments have been added as a supplemental figure (Fig S6) and corresponding text starting at line 198. Indeed, our UbVs are still active against PLpro from the alpha and beta variants. We were not able to include the omicron variant-encoded PLpro in this experiment. However, sequence alignments suggest that the omicron PLpro will also be inhibited by our UbVs. We have made a brief remark on the virus variants in the discussion (line 360 in the revised version).

*Reviewer #3: The authors hypothesize that their inhibitor will affect endogenous ISGylation based on PLPro’s activity as a protease, however they test this in Vero cells which are derived from monkey kidney and have an aberrant interferon response. In addition, unlike ubiquitin ISG15 is not well conserved between metazoan species. I suggest that the authors broaden their work to test the activity of their inhibitor in a human cell line which will express ISG15 and be conjugation competent (since not all cell lines express Ube1L the E1 enzyme for ISG15). The authors could use A549 which ectopically express Ace2 or Calu-3 or even THP-1 cells. With these cell types they could look for the loss of targeting of ISGylation following ectopic expression of the ubiquitin variant or reduced viral load in the lung epithelial cell lines. This could also be done using ISGylated targets taken from cells and subjected to PLPro activity in vitro, which may have more complex chain topology than ISG15-AMC. Ultimately, these experiments will allow them to test inhibition of human ISGylation in lung epithelial cells, which will be more relevant for the implications of their findings.*

Reviewer 3 suggested including additional evidence to support the inhibitory function of our UbVs on the deISGylation activity of SARS-CoV-2 PLpro. This is now included as Fig 2 in the revised manuscript.

**(Part III – Minor Issues)**

*Reviewer #1: 1. The discussion focuses on the potential of Ub variants as inhibitors. However, a second important observation from this study is that blocking a site distal to the active site inhibits PLpro activity, thus identifying an additional target for antiviral drug discovery.
2. Short title: modify to something like Ubiquitin variants identify distal sites for inhibiting SARS-CoV-2 PLpro activity. More experiments using soluble UbVs that can be administered to cells would be needed to say UbVs are antiviral agents. It does seem like this is a future goal for this research.
3.line 649: which isolate of SARS-CoV-2? Was it passaged and re-sequenced (it was it adapted to replication in VeroE6 cells). This may be important later if you start to look at inhibition of VOCs using this system.*

1. Reviewer 1 suggests adding to the discussion that blocking the distal site op PLpro is a novel antiviral drug target. Even though the UbVs do bind to this distal site, they likely exert their inhibitory effects by blocking access of the substrates to the catalytic site. Therefore, we cannot conclude that the distal site itself is an additional target of antiviral drug discovery. Further research will be needed to reveal whether binding to this same distal site by e.g. small chemical compounds also has an inhibitory effect. We have included a clarification pertaining to the steric hindrance caused by the UbVs in the discussion at line 376.

2. Reviewer 1 suggests a modification of the short title. We have now changed this to ‘Ubiquitin variants act as inhibitors of SARS-CoV-2 PLpro’.

3. Lastly, reviewer 1 questions which isolate of SARS-CoV-2 is referred to in line 649. The exact isolate name and description of its passage on Vero E6 cells is mentioned under ‘Cells and virus culture’ in the Materials and Methods section (line 439). For clarity purposes, we have added the isolate name to line 741/742 as well (old line 649). Furthermore, it is important to mention that our isolate was analyzed using NGS. This showed that the mutations in the furin cleavage site (in the spike protein), that are known to arise in Vero cells, were present in less than 2% of the molecules sequenced. In addition, the PLpro protein did not contain mutations relative to the published sequence of the Wuhan virus sequence after limited passaging in Vero cells. We added the latter statement to the materials and methods section in the text to clarify this (line 445).

*Reviewer #2: Minor comments:

- Figure S1 needs a ladder on the gel
- Fig 2C – can the expression level of Ub.AA and UbV be shown
- Please include β-strands in Figure for easy identification of strands
- The authors state that infection was performed in Vero cells. It should be clarified whether these are Vero cells expressing Tmprss2 or Ace2 to make them susceptible to infection.*

To address these minor comments of reviewer 2:

- We have added a ladder to figure S1
- We have verified the expression of the UbV for the experiment pertaining to figure 3C (old figure 2C) using microscopy. As we consider this standard practice before continuing with the experiment, we have not included these microscopy images in the manuscript.
- We have also included labels for the β-strands to the structure for clarity purposes.
- The reviewer asked for clarification of which Vero cells were used for infection. The cells that were used are Vero E6 cells that are naturally susceptible to infection with SARS-CoV-2 (see the following reference: Ogando NS, Dalebout TJ, Zevenhoven-Dobbe JC, Limpens RWAL, van der Meer Y, Caly L, Druce J, de Vries JJC, Kikkert M, Bárcena M, Sidorov I, Snijder EJ. SARS-coronavirus-2 replication in Vero E6 cells: replication kinetics, rapid adaptation and cytopathology. J Gen Virol. 2020 Sep;101(9):925-940. doi: 10.1099/jgv.0.001453. PMID: 32568027; PMCID: PMC7654748). To clarify this in the text, we have adjusted the materials and methods section at line 719 to include that Vero E6 cells are naturally susceptible to infection by SARS-CoV-2 and have included the reference above.
